# Comparative Genomics Revealed Genetic Diversity and Species/Strain-Level Differences in Carbohydrate Metabolism of Three Probiotic Bifidobacterial Species

**DOI:** 10.1155/2015/567809

**Published:** 2015-07-05

**Authors:** Toshitaka Odamaki, Ayako Horigome, Hirosuke Sugahara, Nanami Hashikura, Junichi Minami, Jin-zhong Xiao, Fumiaki Abe

**Affiliations:** Food Science and Technology Institute, Morinaga Milk Industry Co., Ltd., No. 1-83, 5-Chome Higashihara, Zama, Kanagawa, Japan

## Abstract

Strains of *Bifidobacterium longum, Bifidobacterium breve*, and *Bifidobacterium animalis* are widely used as probiotics in the food industry. Although numerous studies have revealed the properties and functionality of these strains, it is uncertain whether these characteristics are species common or strain specific. To address this issue, we performed a comparative genomic analysis of 49 strains belonging to these three bifidobacterial species to describe their genetic diversity and to evaluate species-level differences. There were 166 common clusters between strains of *B. breve* and *B. longum*, whereas there were nine common clusters between strains of *B. animalis* and *B. longum* and four common clusters between strains of *B. animalis* and *B. breve*. Further analysis focused on carbohydrate metabolism revealed the existence of certain strain-dependent genes, such as those encoding enzymes for host glycan utilisation or certain membrane transporters, and many genes commonly distributed at the species level, as was previously reported in studies with limited strains. As *B. longum* and *B. breve* are human-residential bifidobacteria (HRB), whereas *B. animalis* is a non-HRB species, several of the differences in these species' gene distributions might be the result of their adaptations to the nutrient environment. This information may aid both in selecting probiotic candidates and in understanding their potential function as probiotics.

## 1. Background

The genus* Bifidobacterium* has been classified into 48 taxa, including 39 species and nine subspecies [1], three of which (*Bifidobacterium longum*,* Bifidobacterium breve*, and* Bifidobacterium animalis*) are commonly applied as bifidobacterial probiotics in the food industry. Many studies have been performed on the functionality of probiotic bifidobacteria in the host, and the properties and functionality of probiotics have generally been thought to be strain dependent [2, 3]. However, previous reports have indicated that certain characteristics, such as the ability to generate vitamins [4], organic acids [5], and antimicrobial components [6, 7] as well as tolerance to stress [8, 9], are species dependent, although there is a degree of variation among strains. In several cases, it is unclear whether certain characteristics are species common or strain specific.

Recent work has provided genomic information for all bifidobacterial species/subspecies [1, 10]. However, because the number of available genomic sequences from each species was limited in these studies, it is difficult to distinguish whether a number of unique genes are species or strain specific.

To further elucidate bifidobacterial genetic diversity and to evaluate species-level differences, we performed comparative genomic analyses of 49 strains belonging to three bifidobacterial species, or* B. longum*,* B. breve*, and* B. animalis*, which are commonly applied as probiotics in the food industry. The use of a large set of genome sequences based on a relatively large number of strains of each species allowed the identification of pan-genome structures and the elucidation of differences in core genome structures among these species. In the present report, given that bifidobacteria have been reported to utilise many unique metabolic pathways for sugar fermentation [11–19], additional analysis was focused on the carbohydrate transport/metabolism of each species/strain.

## 2. Materials and Methods

### 2.1. Bacterial Strains and Cultivation Conditions

The bacterial strains used in this study and general information about these strains are listed in Table 1. The bifidobacterial strains were obtained from stock cultures maintained in the Morinaga Culture Collection (MCC; Morinaga Milk Industry Co., Ltd., Zama, Japan) and the American Type Culture Collection (ATCC, VA). Each strain was cultivated in Difco Lactobacilli MRS (Becton Dickinson, NJ) supplemented with 0.05% L-cysteine·HCl (Kanto Chemical, Tokyo, Japan) at 37°C for 16 h under anaerobic conditions before DNA extraction. The microorganisms were collected by centrifugation, washed once with sterile saline, resuspended in an equivalent volume of sterile saline, and used as seed cultures for fermentation studies.

### 2.2. Assimilation of Human Milk Oligosaccharides

Modified MRS was prepared by removing glucose from the original components and was supplemented with LNT (Dextra Laboratories Ltd., Reading, UK) at a final concentration of 2%. An aliquot of seed culture from each bifidobacterial strain was then inoculated into 200 *μ*L of the modified MRS, with a final concentration of 1%. Growth of each bifidobacterial strain was measured by absorbance at OD600 after cultivation under anaerobic conditions at 37°C for 24 h and 48 h. Experiments were performed in triplicate.

### 2.3. Genome Sequencing and Bioinformatics Analyses

Genomic DNA was extracted using the DNeasy Blood & Tissue Kit (Qiagen, Valencia, CA) according to the manufacturer's protocol. The preparation of genomic libraries was performed with 1 ng of genomic DNA using the Nextera XT DNA Sample Preparation Kit (Illumina Inc., CA) according to the manufacturer's instructions. After PCR amplification and cleanup, the fragment size distribution of the tagmented DNA was analysed using an Agilent 2100 Bioanalyzer and the High Sensitivity DNA Analysis Kit (Agilent Technologies, Santa Clara, CA). The libraries were sequenced using a MiSeq Personal Sequencing System and the MiSeq Reagent Kit v2 (500 cycles) (Illumina Inc.). Quality trimming and* de novo* assembly of the raw paired-end reads were performed using the CLC Genomics Workbench (v 6.0) software package (CLC bio, Aarhus, Denmark) with default settings, except for contig length (minimum contig length = 2,000 bp). Seventeen genomes that were sequenced* de novo* in this study were automatically annotated using the NCBI Prokaryotic Genome Annotation Pipeline (PGAP) 2.0 and were manually checked as part of the process of genome submission to GenBank. Our sequencing efforts resulted in multiple contigs (Table 1). The datasets for the genome annotations for the other 32 strains published previously were retrieved from the FTP server of NCBI [20].

### 2.4. Pan-Genome Analysis

For all of the 49 bifidobacterial strains included in this study, a pan-genome calculation was performed using the PGAP [21]. The ORF content of each genome was organised into functional gene clusters using the gene family (GF) method. Under the GF method, the total protein sequences of each strain were mixed together, with each gene being marked as a strain identifier. BLASTALL searches were performed among the mixed protein sequences, and the filtered BLAST results were clustered using the Markov Cluster algorithm [22], which has been widely used in other studies on prokaryotic genomes and in programs designed to search for orthologues among multiple strains. For each gene pair in a given cluster, the global match region was no less than 50% of the longer gene protein sequence, and the identity was also no less than 50%. The minimum score value and *E*-value applied in BLAST were 50 and 1e-8. Pan-genome profile analysis, genetic variation analysis of functional genes, and function enrichment analysis of gene clusters were then performed. KEGG Orthology (KO) numbers were assigned by the KEGG Automatic Annotation Server (KAAS) using the bidirectional best-hit method [23].

### 2.5. Phylogenetic Trees

Pan-genome-based phylogenetic trees were generated according to a gene distance matrix, which was calculated based on genes that were absent or present in each strain [21], using UPGMA algorithms in PHYLIP. The sequences of 16S rRNA genes were aligned using the ClustalW alignment tool [24] with default parameters, and phylogenetic trees were constructed using UPGMA algorithms in MEGA6.06 [25]. Bootstrap values were calculated using 500 bootstrap replicates. The supertree was built using FigTree v1.4.0.

### 2.6. Nucleotide Sequence Accession Numbers

The* de novo* sequence and annotation data reported herein have been deposited in GenBank under the accession numbers AVQA00000000-AVQE00000000 and AWFK00000000-AWFV00000000.

## 3. Results and Discussion

### 3.1. Phylogenetic Trees for Each Bifidobacterial Species

Based on the pan-genome profiles and 16S rRNA gene sequences, phylogenetic trees were constructed for each species, using* Gardnerella vaginalis* ATCC 14018 as an outgroup species. Each phylogenetic tree was divided into two clusters. However, the classification of subspecies of certain strains based on the genotypes of the strains was contradictory to that previously defined based on phenotypes (Table 1, Supplementary files 1–3 in Supplementary Material available online at http://dx.doi.org/10.1155/2015/567809).* B. animalis* subsp.* animalis* ATCC 27536 and ATCC 27674 fell into the clade with the type strain of* B. animalis* subsp.* lactis*, whereas B.* longum* subsp.* longum* JDM 301 and* B. longum* subsp.* infantis* 157F fell into the clades with the type strains of* B*.* longum* subsp.* infantis* and B.* longum* subsp.* longum*, respectively. In this study, we adopted subspecies of these strains based on the phylogenetic trees for further analyses.

### 3.2. Core and Pan-Genome Structures

**Figure 1:**
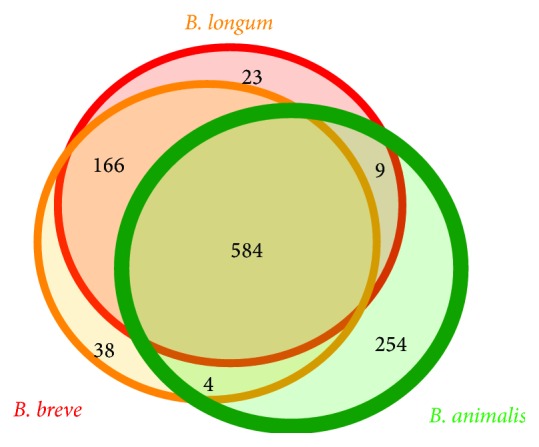
Venn diagram of the homologous clusters shared among the core genes. The surfaces are approximately proportional to the number of genes.

After clustering the functional genes for each species, respective totals of 5,471, 4,053, and 2,833 clusters and 966, 1,221, and 1,092 core clusters were obtained for* B. longum*,* B. breve,* and* B. animalis* (Supplementary file 4). These numbers are similar to those reported previously [11, 26, 27]. However, we identified a wider variety of genomes in* B. longum* compared with a previous report [11]. This result may suggest that* B. longum* has an open pan-genome; that is,* B. longum* may exhibit a robust ability to import new genes, allowing it to adapt to each ecological niche over its long history of evolution. In the current study, to reveal differences among species, a total of 90,442 genes from the 49 strains were jointly clustered. These genes were divided into 8,818 homologous clusters, and there were 584 common clusters among all of the bifidobacterial strains (Figure 1). Additional analysis revealed that 404 of these clusters were commonly clustered with* G. vaginalis* ATCC 14018 as an outgroup species. Therefore, the number of specific gene clusters among the three bifidobacterial species was estimated to be less than 180. There were 166 common clusters between strains of* B. breve* and* B. longum*, whereas there were nine common clusters between strains of* B. animalis* and* B. longum* and four common clusters between strains of* B. animalis* and* B. breve* (Figure 1).

### 3.3. Metabolism of Host Glycans

**Figure 2:**
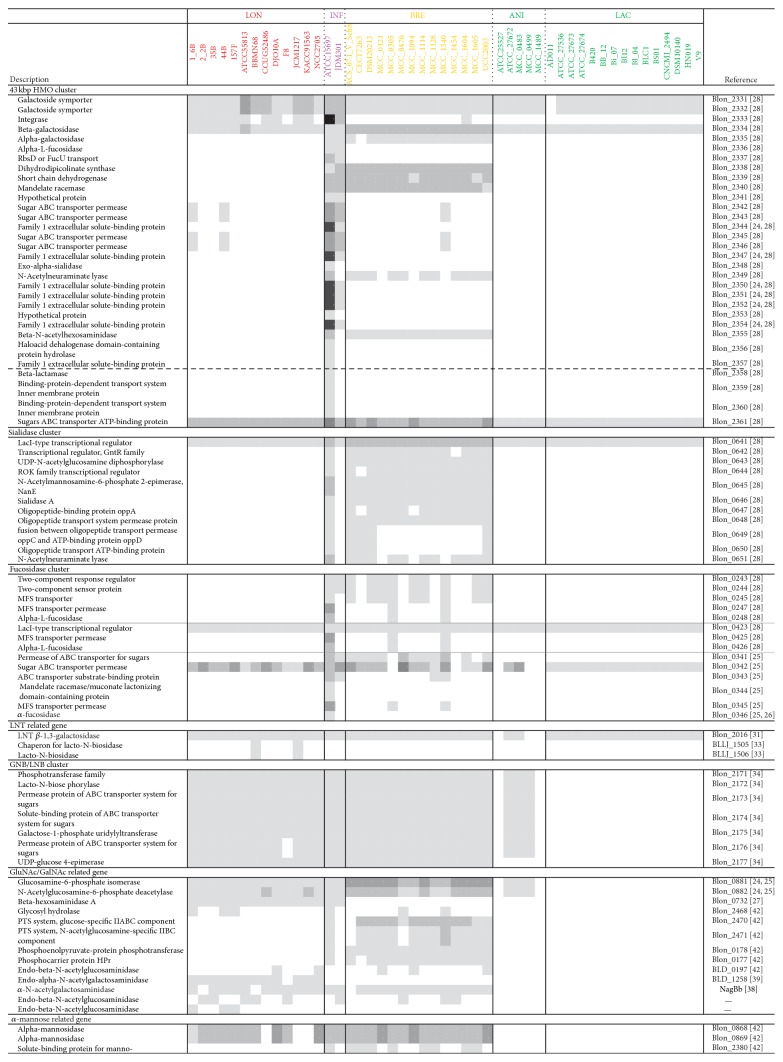
Distribution of genes involved in the metabolism of host glycans, such as human milk oligosaccharides, mucin, and N-glycans. The abbreviations used for species/subspecies are as follows: INF,* B. longum *subsp.* infantis*; LON,* B. longum* subsp.* longum*; BRE,* B. breve*; ANI,* B. animalis *subsp.* animalis*; LAC,* B. animalis *subsp.* lactis.*

Bifidobacteria have been reported to employ many unique metabolic pathways for sugar fermentation to enable them to utilise diverse carbohydrates in the intestine that are not utilised by their hosts [11–19].  Figure 2 shows the distributions of genes involved in the metabolism of host glycans, such as human milk oligosaccharides (HMOs), mucin, and N-glycans.

#### 3.3.1. HMOs

A 43 kb cluster in the genome of* B. longum* subsp.* infantis* ATCC 15697 has been reported to be one of the most characteristic gene clusters for the utilisation of HMOs [28].

Seven homologous genes encoding extracellular solute-binding proteins predicted to bind oligosaccharides (SBP family 1) were found in only two strains of* B. longum *subsp.* infantis, *whereas homologues of the major facilitator superfamily (Blon_2331, 2332) were found in all strains except for* B. animalis *subsp.* lactis* AD011. Two of four gene homologues encoding glycoside hydrolases (alpha-galactosidase and beta-N-acetylhexosaminidase) were found in strains of* B. breve *as well as* B. longum* subsp.* infantis. *Fucosidase (Blon_2336) and sialidase (Blon_2348) homologues were found only in* B. longum *subsp.* infantis. *However, certain strains of* B. breve* possessed other gene clusters related to fucose and sialic acid incorporation (Figure 2).

Regarding the core structure of HMOs, three of the four predominant HMOs (lacto-N-fucopentaose I (LNFP I), lacto-N-difucohexaose I (LNDFH I), and lacto-N-tetraose (LNT)) exhibit the LNT structure. Therefore, the enzyme required for the utilisation of LNT is crucial in the utilisation of HMOs and the colonisation of the infant intestine. Certain strains belonging to* B. bifidum, B. longum* subsp.* longum, B. longum* subsp.* infantis,* and* B. breve *have been reported to act as LNT consumers [29, 30]. The last two species incorporate intact LNT via an unidentified transporter and then hydrolyse it intracellularly using LNT *β*-1,3-galactosidase (Blon_2016) [31]. In our study, using type strains, we confirmed the ability of* B. longum* subsp.* longum, B. longum* subsp.* infantis,* and* B. breve*, but not* B. animalis* subsp.* animalis* and* B. animalis* subsp.* lactis*, in the utilisation of LNT (Supplementary file 5). Unexpectedly, Blon_2016 homologues were also present in* B. animalis* strains. Based on phylogenetic analysis, Yoshida et al. [31] discovered that there is a close homologue of this gene (amino acid identity > 95%) that falls into a clade of strains of infant gut-related species (i.e.,* B. breve*,* B. longum *subsp.* infantis, *and* B. longum *subsp.* longum*), whereas the gene in* B. animalis* was observed to be distant from this clade. These results imply the existence of different substrate specificity for LNT *β*-1,3-galactosidase, although further investigations are necessary to examine this issue.* B. bifidum *and certain strains of* B. longum* subsp.* longum *produce a secretory enzyme, LNBase, that hydrolyses LNT into lacto-*N*-biose (LNB) and lactose [32, 33]. In the present study, homologous genes encoding LNBase (BLLJ_1506) and the chaperone for this enzyme (BLLJ_1505) were found in two strains of* B. longum* subsp.* longum*. The LNBase of* B. longum* subsp.* longum* has been reported to be able to hydrolyse the GlcNAc*β*1-3Gal linkage in LNT, LNFP I, and sialyllacto-N-tetraose. These results imply the existence of different mechanisms for the utilisation of LNT decorated by fucose and sialic acid.* B. bifidum* might digest LNT after releasing its own fucosidase and sialidase, whereas* B. longum* subsp.* longum *lacks these enzymes but can directly degrade decorated LNT.

Subsequently, the liberated LNB is imported into the cells via the galacto-N-biose (GNB)/LNB transporter for further degradation [34]. The disaccharide is then phosphorolysed by a GNB/LNB phosphorylase to produce Gal-1-P and GlcNAc and is further metabolised [34]. In the current study, a seven-gene operon involved in this pathway was observed in nearly all strains of* B. longum *and* B. breve* and in three strains of* B. animalis* subsp.* animalis*. This distribution is in complete accord with results regarding* in vitro* LNB utilisation [35]. A previous report revealing a strong ability to grow in human milk also supports these claims [36].

#### 3.3.2. Mucin and N-Glycans

Another substrate for this metabolic pathway is GNB, which is a core structure of gastrointestinal mucin. Mucins are extensively O-glycosylated proteins and are thought to serve as a potential carbon source for gut microbiota. Studies have revealed that the main core structures of gastric/duodenal and intestinal mucins are core-1, 2-type and core-3-type O-glycans, respectively [37, 38]. A homologous gene encoding *α*-*N*-acetylgalactosaminidase, acting on core-3-type O-glycan (NagBb) [39], was observed in 2 strains of* B. longum* subsp.* infantis*, 11 strains of* B. longum* subsp.* longum,* and 13 strains of* B. breve* in the present study. In contrast, a gene encoding endo-*α*-N-acetylgalactosaminidase, which is predicted to release GNB-containing glycans from core-1-type O-glycan mucin (BLD_1258) [40], was observed only in* B. longum* subsp.* longum*. In addition, the terminal ends of glycoconjugates in the suckling gut have been reported to predominantly consist of sialic acid, whereas in the adult, they predominantly consist of fucose [41, 42]. In our study, nearly all strains of* B. longum* subsp.* infantis *and* B. breve* contained a gene encoding sialidase, which might be useful for this species to colonise the infant intestine.

Most strains of* B. longum *and* B. breve*, but not* B. animalis *strains, were found to possess genes involved in the pathway for the utilisation of N-acetylglucosamine (GlcNAc) and GalNAc, which are the major constituents of host glycans. Furthermore, gene homologues encoding endo-beta-N-acetylglucosaminidase (BLD_0197) [43], which releases complex N-glycans from human milk glycoproteins, and alpha-mannosidase (Blon_0868, 0869) were found in certain strains of these two species. These enzymes involved in the degradation of host glycans might play a role in the utilisation of intrinsic carbohydrate sources.

Bifidobacteria are generally residents of the intestines of animals, including warm-blooded mammals and social insects, and several bifidobacterial species are typical inhabitants of the human gut (designated human-residential bifidobacteria or HRB). All strains of* B. longum *and* B. breve,* which are typical HRB, possess certain genes related to the pathway for the utilisation of LNT/LNB, which are core structures of type I oligosaccharides that are specific to human breast milk (Figure 2). Among HRB strains in our study, nearly all possessed gene operons involved in the GNB/LNB pathway, whereas genes upstream of HMO utilisation, such as fucosidase, sialidase, and LNBase, were species/strain dependent. These results suggest that each HRB strain might evolve to assimilate HMOs, for which hundreds of types of structures have been reported [44]. In contrast,* B. animalis* is a non-HRB species; although certain strains of* B. animalis* subsp.* lactis* have also been isolated from humans, they have been found in faecal samples but rarely in colon mucosal samples [27, 45]. In addition, previous reports have shown that* B. animalis* is a strictly monophyletic group, and the evolutionary distance between* B. animalis* and species of HRB was shown to be relatively far based on 16S rRNA multigene alignments and comparative genomics [11, 13, 27, 46]. Our results indicate that there are fewer homologues involved in the degradation of host glycan by* B. animalis *(Figure 2). These results suggest that the observed differences in the gene distribution might be the result of the adaptation of these strains to their residential environments.

### 3.4. Carbohydrate-Active Enzymes for Plant-Derived Sugars

**Figure 3:**
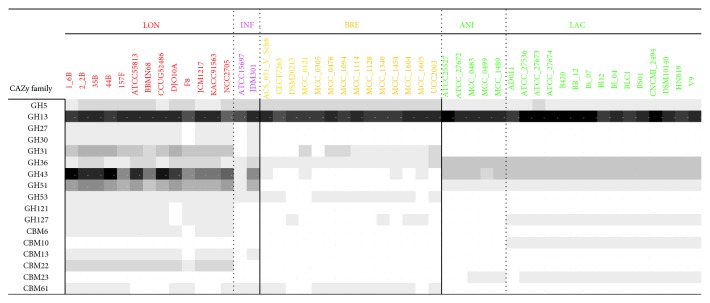
Distribution of genes involved in the metabolism of plant-derived sugars predicted by the CAZy database. The abbreviations used for species/subspecies are as follows: INF,* B. longum *subsp.* infantis*; LON,* B. longum* subsp.* longum*; BRE,* B. breve*; ANI,* B. animalis *subsp.* animalis*; LAC,* B. animalis *subsp.* lactis.*

Regarding extrinsic carbohydrates, a variety of resistant fibres, which can be dietary compounds, are delivered to the colon, where bifidobacteria reside. Based on CAZy classification, all of the strains possessed a large number of homologous genes encoding GH 13 family members (Figure 3), which has previously been reported as a characteristic feature of bifidobacterial genomes [47, 48]. These enzymes are typical enzymes for the degradation of alpha-glucopyranose units such as pullulan, starch, and amylopectin. In particular,* B. longum* subsp.* longum* was shown to be genetically well equipped for the fermentation of plant-derived sugars (Figure 3), which are assumed to be not introduced into the infant gut before weaning. A large number of GH 43 and 51 family members, which are enzymes responsible for the degradation of arabinose/xylose units such as arabinofuranoside and xylan, were found to be specific to* B. longum* subsp.* longum*, with strain specificity for several of these genes. Taken together with the distribution of genes for host glycan utilisation, such a wide range of genes for carbohydrate utilisation provides an advantage for colonisation of the human intestine by* B. longum* subsp.* longum*. This result might explain why only* B. longum* subsp.* longum* is a predominant species in both the infant and the adult intestines [49].

### 3.5. Membrane Transporters for Carbohydrate

KO assignment indicated several differences in the distribution of carbohydrate transporters at the subspecies level. The subspecies that possessed the greatest number of transporter homologues was* B. longum* subsp.* infantis* (83.5 ± 2.1), including taxon-specific transporters for GlcNAc, phosphonate, and urea, whereas* B. animalis* subsp.* lactis* possessed the lowest number of these homologues (33.7 ± 1.3). All three species can assimilate fructose; however, only species of* B. longum* exhibited a set of high-affinity fructose-specific ABC transporters (K02056, K02057, and K02058), which have been reported to be involved in efficiently converting fructose to acetate under certain gut conditions [5].* B. longum* subsp.* longum* lacked a homologous gene set for arabinogalactan transport. However, twelve strains of* B. longum* subsp.* longum* possessed a set of homologous genes encoding extracellular enzymes for the degradation of arabinogalactan (BLLJ_1840) [50].* B. breve *also exhibited certain unique ABC transporters for lactose/L-arabinose, ribose, and sn-glycerol 3-phosphate.

**Figure 4:**
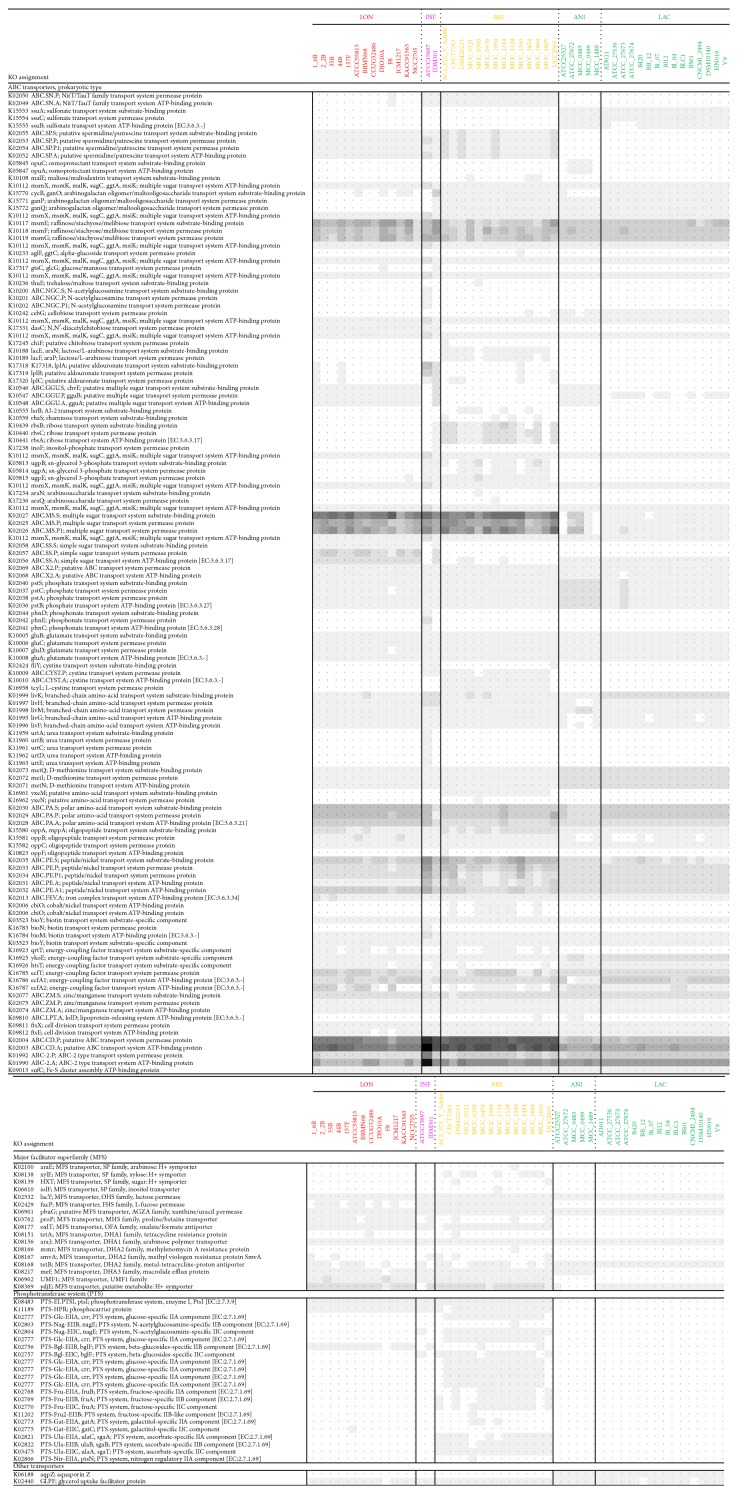
Distribution of genes encoding membrane transporters for carbohydrate. The abbreviations used for species/subspecies are as follows: INF,* B. longum *subsp.* infantis*; LON,* B. longum* subsp.* longum*; BRE,* B. breve*; ANI,* B. animalis *subsp.* animalis*; LAC,* B. animalis *subsp.* lactis.*

We found distinctive differences between the three species in terms of the distribution of genes related to the phosphotransferase system (PTS), which is known to be a multicomponent system specific for sugar uptake that operates in global carbon regulation in many bacteria [51]. Strains of* B. longum* and* B. breve* possessed three homologues, encoding phosphoenolpyruvate-protein kinase, phosphocarrier protein, and beta-glucoside-PTS, whereas none of the* B. animalis* strains exhibited these genes. Additionally, nearly all strains of* B. breve *possessed homologues of N-acetylglucosamine, glucose, fructose, and ascorbate, which are involved in the PTS (Figure 4).

## 4. Conclusions

Certain strains belonging to the three bifidobacterial species targeted in this study have been reported to exert numerous beneficial effects on human health as probiotics. Probiotic functionality is generally thought to be strain dependent. In fact, our analysis confirmed that certain genes, such as those encoding membrane transporters and enzymes for host glycan utilisation, are strain dependent. However, our data also demonstrated that there are common characteristics in each species that may be important in light of the species' health-promoting effects on their hosts. Additionally, differences in characteristics might be a result of adaptation to the nutrient environments of each species (such as HRB versus non-HRB). Our results support previous observations based on the investigation of certain type strains of bifidobacterial species and enable the qualification of several of these characteristics as species common or strain specific [33, 39, 50, 52]. Taken together with other characteristics, such as vitamin metabolism [4, 53], colonisation factors [54], and extracellular components [55, 56], we believe that these findings will help to predict the features of probiotic strains. However, further study is needed to evaluate other non-HRB and HRB bifidobacterial species to attain a better understanding of the characteristics of these bacteria as well as the mechanisms underlying their residence in the host intestine and their potential functions as probiotics.

## Supplementary Material

These files indicate the phylogenetic trees to show the contradictory results based on phenotype and genotype.

## Figures and Tables

**Table 1:** General genome features of *Bifidobacterium* species included in this study.

Species	Genotype based on		Strain	Status	Chromosome	Plasmid	Contig	Length (kbp)	CDSs	Source	GenBank accession number
	16SrRNA	Whole genes									
*B. longum *ssp. *infantis *	*infantis *	*infantis *	ATCC 15697	Complete	1			2,833	2,416	Human infant faeces	CP001095
*B. longum *ssp. *longum *	*infantis *	*infantis *	JDM301	Complete	1			2,478	1,958	Human vagina	CP002010
*B. longum *ssp. *longum *	*longum *	*longum *	BBMN68	Complete	1			2,266	1,804	Human elderly faeces	CP002286
*B. longum *ssp. *infantis *	*longum *	*longum *	157F	Complete	1	2		2,409	1,999	Human infant faeces	AP010890, AP010891, and AP010892
*B. longum *ssp. *longum *	*longum *	*longum *	JCM 1217	Complete	1			2,385	1,924	Human infant faeces	AP010888
*B. longum *ssp. *longum *	*longum *	*longum *	KACC 91563	Complete	1	2		2,396	1,985	Human infant faeces	CP002794, CP002795, and CP002796
*B. longum *	*longum *	*longum *	NCC2705	Complete	1	1		2,260	1,728	Human infant faeces	AE014295 and AF540971
*B. longum *	*longum *	*longum *	DJO10A	Complete	1	2		2,390	2,000	Human adult faeces	CP000605, AF538868, and AF538869
*B. longum *ssp. *longum *	*longum *	*longum *	F8	Complete	1			2,385	1,681	Human feces	FP929034
*B. longum *ssp. *longum *	*longum *	*longum *	ATCC 55813	Unfinished			140	2,373	2,109	Human infant faeces	ACHI00000000
*B. longum *ssp. *longum *	*longum *	*longum *	CCUG 52486	Unfinished			55	2,453	2,240	Human elderly faeces	ABQQ00000000
*B. longum *ssp. *longum *	*longum *	*longum *	1-6B	Unfinished			171	2,686	2,425	Human children feces	AJTF00000000
*B. longum *ssp. *longum *	*longum *	*longum *	2-2B	Unfinished			141	2,625	2,412	Human children feces	AJTJ00000000
*B. longum *ssp. *longum *	*longum *	*longum *	35B	Unfinished			131	2,514	2,260	Human infant faeces	AJTI00000000
*B. longum *ssp. *longum *	*longum *	*longum *	44B	Unfinished			62	2,559	2,262	Human infant faeces	AJTM00000000

*B. breve *	*breve *	*breve *	ACS-071-V-Sch8b	Complete	1			2,327	1,826	Human vagina	CP002743
*B. breve *	*breve *	*breve *	UCC2003	Complete	1			2,423	1,852	Human infant faeces	CP000303
*B. breve *	*breve *	*breve *	DSM 20213	Unfinished			117	2,298	2,263	Human infant faeces	ACCG00000000
*B. breve *	*breve *	*breve *	CECT 7623	Unfinished			34	2,314	1,725	Human milk	AFVV00000000
*B. breve *	*breve *	*breve *	MCC0121	Unfinished			27	2,436	2,079	Human infant faeces	AVQA00000000
*B. breve *	*breve *	*breve *	MCC0305	Unfinished			22	2,287	1,927	Human adult faeces	AWFR00000000
*B. breve *	*breve *	*breve *	MCC0476	Unfinished			15	2,234	1,851	Human adult faeces	AVQB00000000
*B. breve *	*breve *	*breve *	MCC1094	Unfinished			23	2,327	1,944	Human infant faeces	AWFS00000000
*B. breve *	*breve *	*breve *	MCC1114	Unfinished			33	2,487	2,136	Human infant faeces	AVQC00000000
*B. breve *	*breve *	*breve *	MCC1128	Unfinished			25	2,480	2,143	Human infant faeces	AVQD00000000
*B. breve *	*breve *	*breve *	MCC1340	Unfinished			23	2,373	1,986	Human infant faeces	AWFT00000000
*B. breve *	*breve *	*breve *	MCC1454	Unfinished			14	2,457	2,111	Human infant faeces	AWFU00000000
*B. breve *	*breve *	*breve *	MCC1604	Unfinished			15	2,206	1,830	Human elderly faeces	AVQE00000000
*B. breve *	*breve *	*breve *	MCC1605	Unfinished			37	2,324	1,976	Human elderly faeces	AWFV00000000

*B. animalis *ssp. *animalis *	*animalis *	*animalis *	ATCC 25527	Complete	1			1,933	1,538	Rat feces	CP002567
*B. animalis *ssp. *animalis *	*animalis *	*animalis *	ATCC 27672	Unfinished			9	1,990	1,532	Rat feces	AWFQ00000000
*B. animalis *ssp. *animalis *	*animalis *	*animalis *	MCC0483	Unfinished			27	2,176	1,750	Rat feces	AWFK00000000
*B. animalis *ssp. *animalis *	*animalis *	*animalis *	MCC0499	Unfinished			11	2,134	1,721	Rat feces	AWFN00000000
*B. animalis *ssp. *animalis *	*animalis *	*animalis *	MCC1489	Unfinished			14	1,910	1,474	Guinea pig feces	AWFO00000000
*B. animalis *ssp. *lactis *	*lactis *	*lactis *	AD011	Complete	1			1,934	1,527	Human infant faeces	CP001213
*B. animalis *ssp. *animalis *	*lactis *	*lactis *	ATCC 27536	Unfinished			18	1,912	1,561	Chicken feces	AWFL00000000
*B. animalis *ssp. *animalis *	*lactis *	*lactis *	ATCC 27673	Unfinished			21	1,937	1,576	Sewage	AWFP00000000
*B. animalis *ssp. *animalis *	*lactis *	*lactis *	ATCC 27674	Unfinished			18	1,912	1,561	Rabbit feces	AWFM00000000
*B. animalis *ssp. *lactis *	*lactis *	*lactis *	B420	Complete	1	1		1,939	1,561	No information	CP003497
*B. animalis *ssp. *lactis *	*lactis *	*lactis *	BB-12	Complete	1			1,942	1,642	Yoghurt	CP001853
*B. animalis *ssp. *lactis *	*lactis *	*lactis *	Bi-07	Complete	1			1,939	1,597	No information	CP003498
*B. animalis *ssp. *lactis *	*lactis *	*lactis *	Bl-04	Complete	1			1,939	1,567	Human infant faeces	CP001515
*B. animalis *ssp. *lactis *	*lactis *	*lactis *	Bl12	Complete	1			1,938	1,518	Colonoscopic sample	CP004053
*B. animalis *ssp. *lactis *	*lactis *	*lactis *	BLC1	Complete	1			1,944	1,518	No information	CP003039
*B. animalis *ssp. *lactis *	*lactis *	*lactis *	BS01	Unfinished			7	1,932	1,572	No information	AHGW00000000
*B. animalis *ssp. *lactis *	*lactis *	*lactis *	CNCM I-2494	Complete	1			1,943	1,660	No information	CP002915
*B. animalis *ssp. *lactis *	*lactis *	*lactis *	DSM 10140	Complete	1			1,938	1,565	Yoghurt	CP001606
*B. animalis *ssp. *lactis *	*lactis *	*lactis *	HN019	Unfinished			28	1,916	1,578	No information	ABOT00000000
*B. animalis *ssp. *lactis *	*lactis *	*lactis *	V9	Complete	1			1,944	1,572	Human infant faeces	CP001892
