# Supplementary material for: Comparative Genomics Revealed Genetic Diversity and Species/Strain-Level Differences in Carbohydrate Metabolism of Three Probiotic Bifidobacterial Species
Source: Int J Genomics. 2015 Jul 5;2015:567809. doi: 10.1155/2015/567809 (PMC4506816; doi:10.1155/2015/567809)
Supplement: Supplementary file 1 — These files indicate the phylogenetic trees to show the contradictory results based on phenotype and genotype. [file 567809.f1.pdf]

Supplementary file 1 – Phylogenetic tree based on (a) 16S rRNA gene sequences and (b) whole genes of *B. longum*

Each phylogenetic tree was constructed using (a) the ClustalW program and (b) the PGAP pipeline [21] via the neighbour-joining method. The numbers in parentheses indicate the (A) locus_tag and (B) accession number

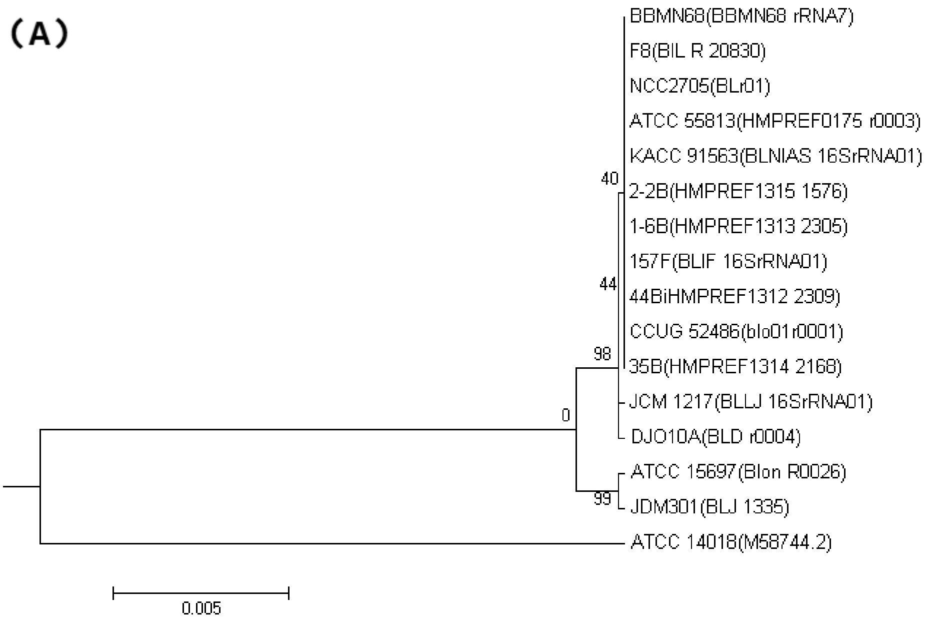

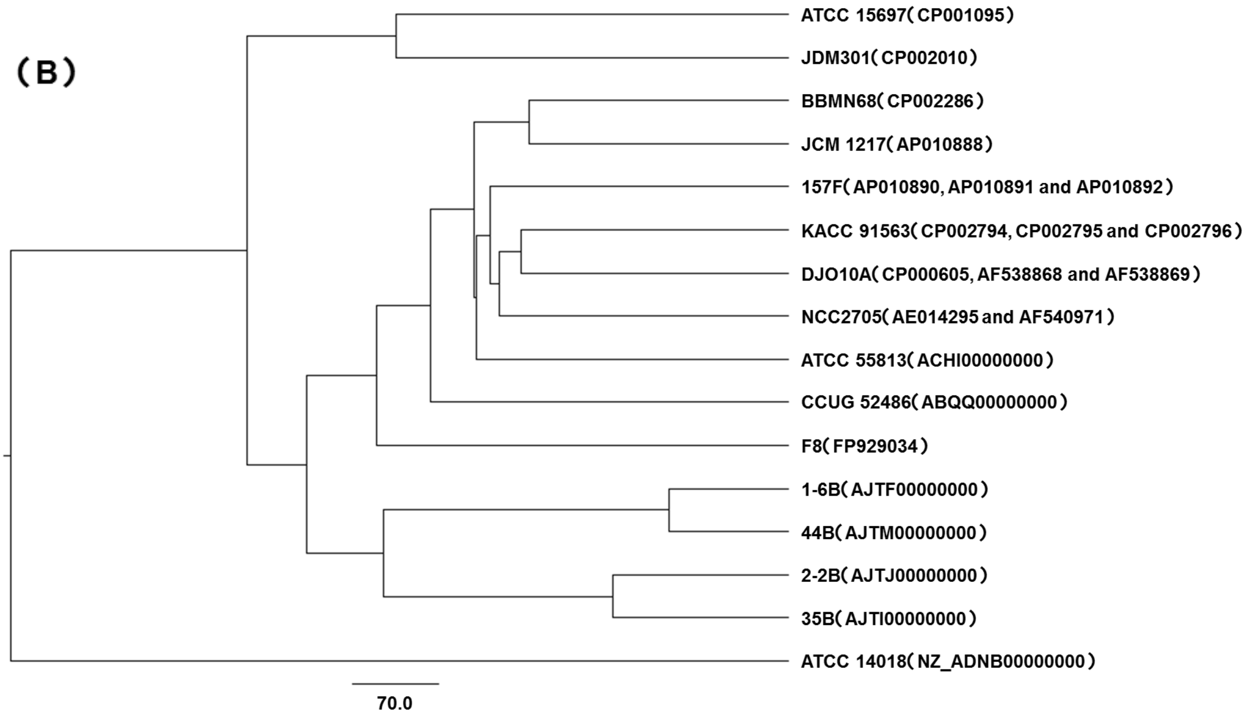

Supplementary file 2 – Phylogenetic tree based on (a) 16S rRNA gene sequences and (b) whole genes of *B. breve*

Each phylogenetic tree was constructed using (A) the ClustalW program and (B) the PGAP pipeline [21] via the neighbour-joining method. The numbers in parentheses indicate the (A) locus_tag and (B) accession number.

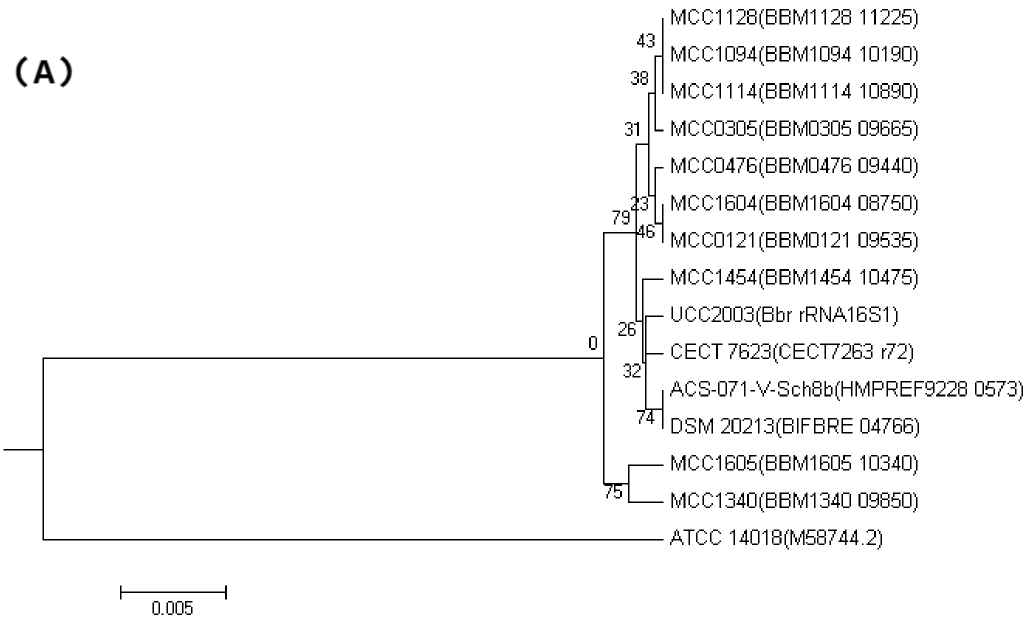

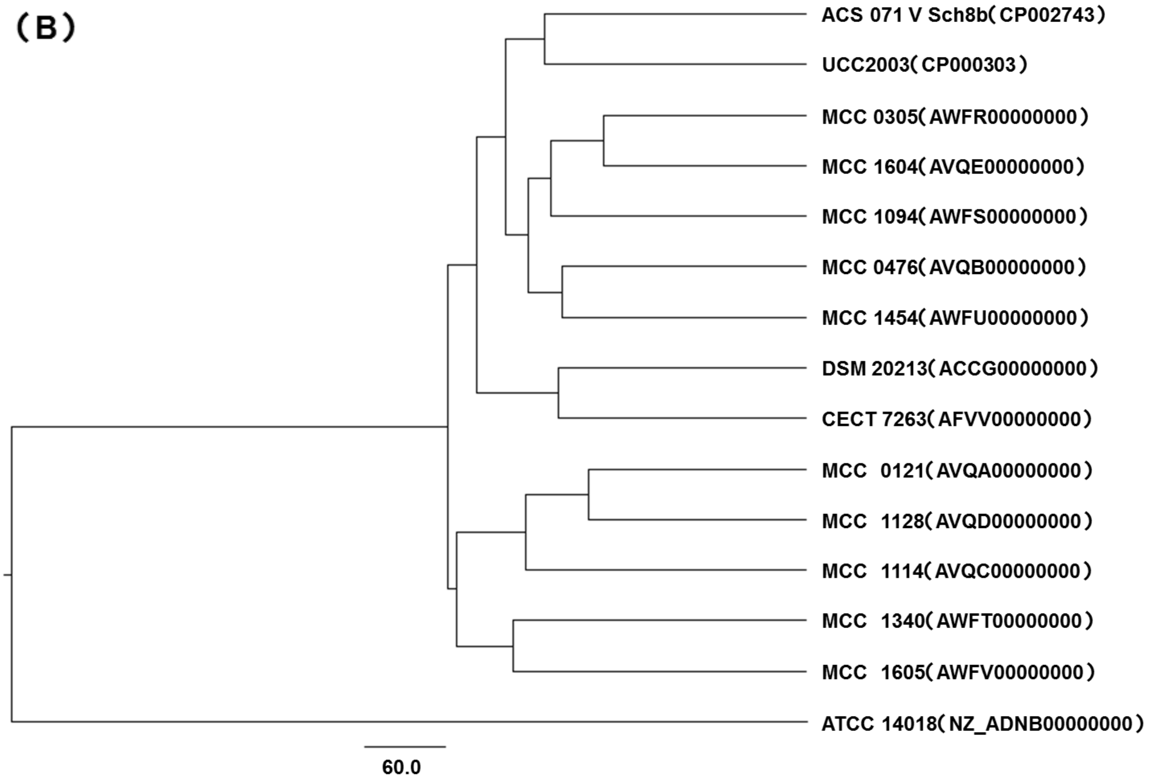

Supplementary file 3 – Phylogenetic tree based on (a) 16S rRNA gene sequences and (b) whole genes of *B. animalis*

Each phylogenetic tree was constructed using (A) the ClustalW program and (B) the PGAP pipeline [21] via the neighbour-joining method. The numbers in parentheses indicate the (A) locus_tag and (B) accession number.

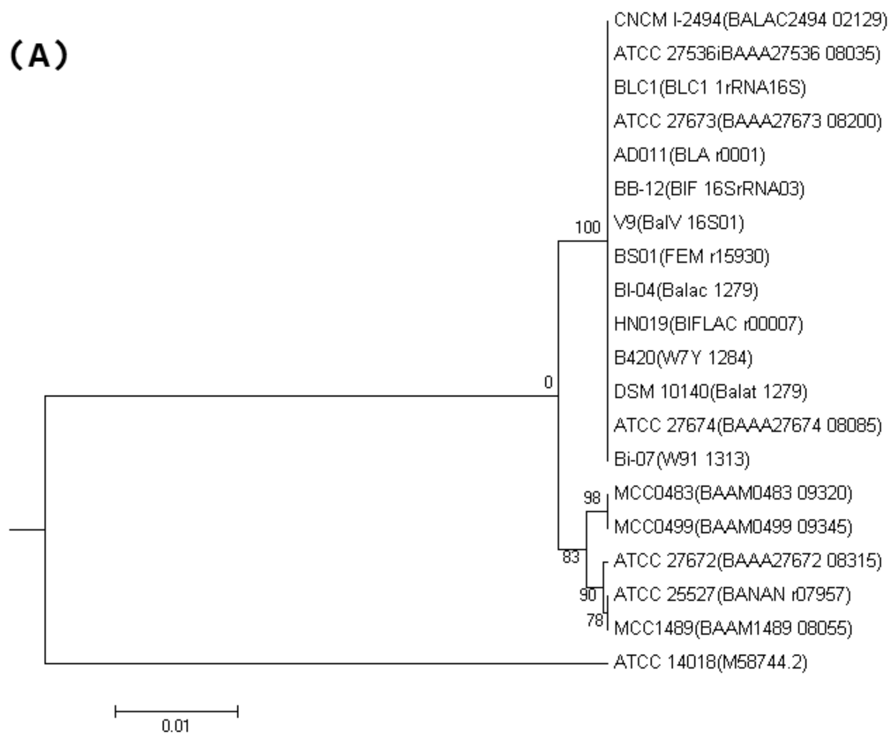

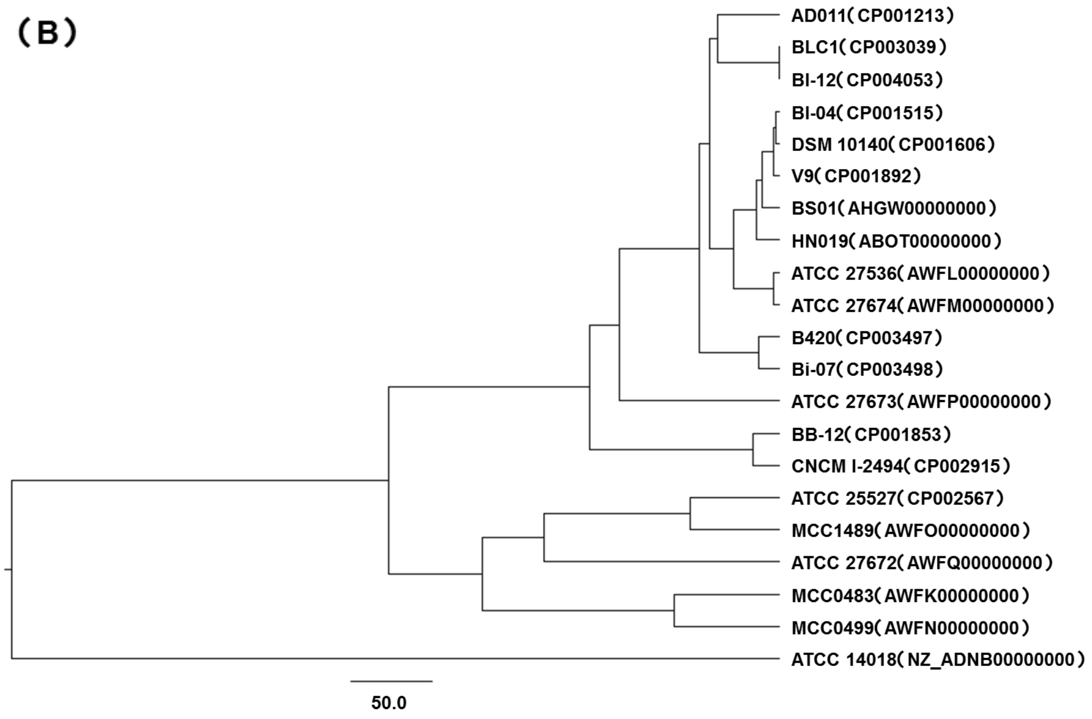

Supplementary file 4 – Pan- and core genome plots for the three analysed species

Solid and open symbols indicate the numbers of pan-genomes and core genomes, respectively. Each colour indicates a different species: red, *B. longum*; orange, *B. breve*; green, *B. animalis*.

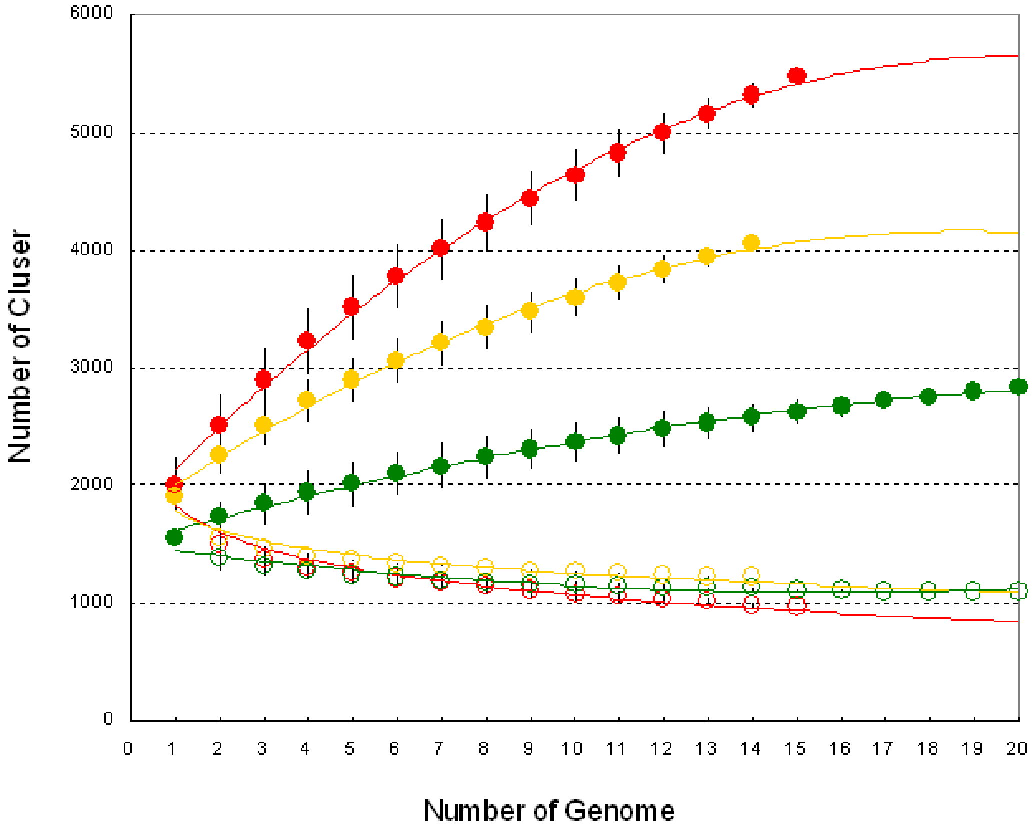

Supplementary file 5 - Ability of bifidobacteria in the assimilation of human milk oligsaccharide, lacto-N-tetraose (LNT)

Species	Strain	24 h		48 h	
		Mean	SD	Mean	SD
B. longum subsp. *longum*	JCM 1217^{T}	1.3	0.03	1.6	0.19
B. longum subsp. *infantis*	ATCC 15697 ^{T}	1.7	0.03	2.0	0.03
B. breve	DSM 20213^{T}	1.5	0.03	2.0	0.14
B. animalis subsp. *animalis*	ATCC 25527^{T}	0.0	0.00	0.0	0.00
B. animalis subsp. *lactis*	DSM 10140^{T}	0.0	0.00	0.0	0.00

OD600 after cultivation at 37ºC under anaerobic conditions for 24 to 48 h in a medium containing 2% of LNT as the sole carbohydrate
